# Employing quality control and feedback to the EQ-5D-5L valuation protocol to improve the quality of data collection

**DOI:** 10.1007/s11136-016-1445-9

**Published:** 2016-10-31

**Authors:** Fredrick Dermawan Purba, Joke A. M. Hunfeld, Aulia Iskandarsyah, Titi Sahidah Fitriana, Sawitri S. Sadarjoen, Jan Passchier, Jan J. V. Busschbach

**Affiliations:** 1 Department of Psychiatry, Section Medical Psychology and Psychotherapy, Erasmus MC University Medical Center Rotterdam, s-Gravendijkwal 230, 3015 CE Rotterdam, The Netherlands; 2 Department of Developmental Psychology, Faculty of Psychology, Padjadjaran University, Jatinangor, Indonesia; 3 Department of Clinical Psychology, Faculty of Psychology, Padjadjaran University, Jatinangor, Indonesia; 4 Center of Applied Psychometrics, Faculty of Psychology, YARSI University, Jakarta, Indonesia; 5 Department of Clinical Psychology, VU University, Amsterdam, The Netherlands

**Keywords:** EQ-5D-5L, Valuation study, Indonesia, Interviewing problem and solution, Quality control

## Abstract

**Objectives:**

In valuing health states using generic questionnaires such as EQ-5D, there are unrevealed issues with the quality of the data collection. The aims were to describe the problems encountered during valuation and to evaluate a quality control report and subsequent retraining of interviewers in improving this valuation.

**Methods:**

Data from the first 266 respondents in an EQ-5D-5L valuation study were used. Interviewers were trained and answered questions regarding problems during these initial interviews. Thematic analysis was used, and individual feedback was provided. After completion of 98 interviews, a first quantitative quality control (QC) report was generated, followed by a 1-day retraining program. Subsequently individual feedback was also given on the basis of follow-up QCs. The Wilcoxon signed-rank test was used to assess improvements based on 7 indicators of quality as identified in the first QC and the QC conducted after a further 168 interviews.

**Results:**

Interviewers encountered problems in recruiting respondents. Solutions provided were: optimization of the time of interview, the use of broader networks and the use of different scripts to explain the project’s goals to respondents. For problems in interviewing process, solutions applied were: developing the technical and personal skills of the interviewers and stimulating the respondents’ thought processes. There were also technical problems related to hardware, software and internet connections. There was an improvement in all 7 indicators of quality after the second QC.

**Conclusion:**

Training before and during a study, and individual feedback on the basis of a quantitative QC, can increase the validity of values obtained from generic questionnaires.

## Introduction

The EQ-5D instrument is a generic health questionnaire developed by the EuroQol Group and widely used to measure health outcomes. The EuroQol Group released the newer version of EQ-5D in 2011, consisting of five levels of severity in each dimension [1]. Several valuation studies of this new questionnaire were conducted internationally with the aim of developing country-specific algorithms for EQ-5D-5L [2–7]. For such valuation studies, the EuroQol Group promotes a standardized protocol: the EQ Valuation Technology, or EQ-VT. This EQ-VT protocol includes a computer supported time trade-off (TTO) exercise, a visual analog scale (VAS) and a discrete choice experiment (DCE) [8]. In earlier TTO, VAS and DCE administrations researchers noticed problems with the quality of the responses such as “non-traders” (those not willing to trade life years for health) and illogical answers, both of which could affect the quality of the data [9]. Another problem has been in obtaining values below the value of dead [10]. One of the reasons the EQ-VT was developed was to overcome such problems [11].

In addition to these efforts, several investigations were conducted in the area of methodology [8, 12], approaches and techniques [13–15] and data analysis [16, 17], in order to increase the quality of the data collection and data reporting, but none of these investigations focused on the interviewers. Such a focus is warranted, since training for interviewers, before and during data collection, has been shown to relate to data quality [9]. However, it is not yet clear how this training is undertaken in a valuation procedure, such as the EQ-VT protocol, nor what are the results of such training. The EuroQol Group has recognized the quality of data collection as a relevant topic and has developed a continuous quality monitor for data collection: the quality control (QC) report tool. How interviewers and supervisors improve in response to this QC report is as yet unknown.

The main purposes of this study were (1) to describe the problems of the interviewers. First of all, finding sufficient subjects who are representative for the general population, the problems interviewers encountered during their interviews and the problems they perceived in their respondents as these respondents endeavored to undertake the TTO, DCE and VAS exercises; (2) to evaluate quantitatively the improvement in interviewers’ skills displayed after a retraining program based on the QC provided by the EuroQol Group office, individual feedback and the advice from the daily supervisor.

## Methods

### Respondents and interviewers

This study is part of a larger valuation study using a health-related quality of life measurement tool, EQ-5D-5L, for the general Indonesian population. Thirteen interviewers were hired to interview 1000 respondents in three different cities (Jakarta, Bandung and Jogjakarta) and their surrounding areas. Quota sampling was used to make the sample representative of the Indonesian population with regard to six demographic characteristics: location (urban–rural), gender, age, level of education, religion and ethnicity [18]. The majority of the interviewers had Bachelors’ degrees in various disciplines, especially quality of life-related majors (e.g., Psychology, Management, Development Communication, Economics). One interviewer had obtained a Master’s degree in Psychology. Each interviewer was included as a participant if she/he fulfilled the following inclusion criteria: present at the first training session and at a retraining program, and completion of at least 15 interviews after retraining.

### Instruments

#### EQ-5D-5L valuation technology (EQ-VT)

To generate national health state values for EQ-5D-5L, and to standardize EQ-5D-5L valuation studies, the EuroQol Group developed a valuation protocol [8] and the EQ-VT computer program. The protocol consists of five parts: (1) general welcome; (2) introduction to the EQ-5D-5L descriptive system, the VAS and the socio-demographic background questions; (3) composite time trade-off (cTTO) tasks [19]; (4) DCE tasks; and (5) round up. All steps were accomplished using computer-assisted face-to-face interviews employing the EQ-VT software provided by the EuroQol Group.

#### Open-ended questionnaire

An open-ended questionnaire comprising three questions was given to the interviewers. (1) “What were the problems that you as an interviewer faced whilst conducting EQ-5D-5L valuation study interviews?” (2) “What were the problems that you think your respondents faced in completing EQ-5D-5L valuation study interviews?” (3) “What were the solutions that you applied to overcome your problems as an interviewer and the problems of your respondents?”.

#### Quality control (QC) report

The QC report takes the form of a Microsoft Excel file that provides a number of statistics related to the quality of the data collected so far, differentiated per interviewer. It measured interviewers’ compliance with the valuation study protocol. Seven protocol compliance indicators were used:

1. The number of health states given a value of zero in the TTO tasks over all the interviews.
2. The mean number of iterative TTO steps used in the wheelchair example by the interviewer, over all his or her interviews. More steps used means the interviewer explained the wheelchair example more thoroughly than less amount of steps.
3. The number of times when a respondent had an inconsistency where the TTO rating of state “55555” was not rated as the state with the lowest value and at least 0.5 higher than the state with the lowest value. If such an inconsistent response was found, the whole interview was deemed to be of low quality and as such “flagged.”
4. The number of times when the duration of time an interviewer used to explain the “wheelchair example” preceding the actual valuation task was less than 180 s. The interview was flagged as being of low quality.
5. Interview duration: the minutes taken to complete the TTO valuation tasks. If the TTO tasks lasted less than 5 min, the interview was flagged.
6. Wheelchair lead time: the interviewer was required to explain the worse-than-dead element of the wheelchair example. If not then the interview was flagged.
7. The overall indicator of quality was the percentage of flagged interviews per interviewer. It was considered that this should be below 40%. The daily supervisor used this indicator as the starting point for the conversation with the interviewer during feedback sessions.

### Training and retraining

The primary training of the interviewers comprised of three sessions: (1) introduction of related concepts, such as health-related quality of life and EQ-5D-5L as a generic questionnaire used to value health states, (2) explanation of the EQ-VT protocol and interviewer instructions and (3) practice in groups.

After 98 interviews, the first quality control report was received based on the protocol compliance indicators. The overall indicator of quality, expressed by the percentage of flagged interviews, was 53%. This was deemed to be quite disappointing by the team, since this should be below 40%. Hence the decision was made to discard the first 98 interviews and hold further sampling until the interviewers were retrained, using the feedback from the quality control report.

All interviewers were invited to the retraining program. The retraining program led by the daily supervisor (FDP) was held in each center and was attended by interviewers from that center only. First, FDP presented the QC report to show overall quality of each center’s interviews based on seven objective indicators of compliance to the protocol. FDP then presented the compliance data of each interviewers. The interviewer explained their difficulties to meet each indicator and provide suitable solutions to overcome these problems. A list of these problems and solutions was made in each center and shared to other centers.

To ensure interviewers adhere every indicators of protocol compliance after the retraining program, FDP created QC reports once in two days. He made notes at a group level and on an individual level and sent this feedback to the interviewers, so that they were able to learn from their own and other interviewers’ performance.

### Procedure

The valuation study was approved by the Health Research Ethics Committee, Faculty of Medicine, Padjadjaran University, Indonesia. The daily supervisor (FDP) created two QC reports using the Quality Control Microsoft Excel file provided by the EuroQol Group office. The first report generated on March 16, 2015 consists of 98 interviews conducted by the interviewers. This report was used as a basis for a retraining program held March 21–24, 2015. The second report generated on May 18, 2015 consists of 168 interviews conducted after the retraining program. On May 18, 2015, the FDP sent an open-ended questionnaire by e-mail to each interviewer, asking them to return the questionnaire within one week. Three days later, all interviewers had sent their answers.

### Data collection and analysis

The data regarding problems encountered by interviewers and respondents, and solutions applied to overcome these problems were considered as qualitative data and analyzed using a thematic analysis approach in order to provide relevant themes. The guidelines of Braun and Clarke [20] were and a qualitative software program, NVivo, was utilized. The first author (FDP) read all the answer documents from the interviewers and built an initial coding directory. Using this initial directory, FDP and two groups consisting of two coders (from the interviewers) each coded the transcripts separately. FDP and one group of coders coded that part of the interviewers’ answers regarding problems encountered by interviewers and the solutions applied. FDP and the second group of coders discussed the other part of the data, the problems perceived by the interviewers as encountered by respondents and the solutions applied. During the coding process, coders frequently contacted the corresponding interviewer to clarify any unclear answer. A discussion was held to achieve agreement on differences that occurred in the initial coding. Codes were collated into potential themes, reviewed by FDP and the other coders, to generate a thematic map of the analysis. Finally, a discussion was held to produce definitions and names for each theme (the coding tree is available upon request). The thematic map, themes and sub-themes’ names and definition were discussed with all interviewers for additional comments. Frequencies for each theme and sub-theme were calculated, and typical citations were noted.

Data from two QC reports regarding interviewers’ performance were treated as quantitative data and analyzed using quantitative statistics software. To analyze the improvement in performance of the interviewers before and after the retraining program, software program SPSS version 21 for Windows was utilized. The Wilcoxon signed-rank test was used to assess the improvement in interviewers’ performance, based on 266 respondents’ data (98 respondents before and 168 respondents after the retraining) on the seven previously mentioned indicators.

## Results

### Interviewer characteristics

In total, 11 out of 13 interviewers were eligible to participate and returned their answers. One interviewer conducted a limited number of interviews in the second wave (below 15) and thus failed to fulfill the inclusion criteria. The other excluded interviewer was not present at the first training session and did not interview any respondent prior to the retraining program. As shown in Table 1, there were 2 male and 9 female interviewers. The interviewers’ ages ranged from 21 to 27 years. The majority were Moslems and the rest Christians. Ethnicity also varied, namely Batak, Minang, Jawa, Sunda, Nusa Tenggara and Ambon. All had a Bachelor’s degree or a higher degree.

**Table 1:** Interviewer demographics (*n* = 11)

Variable	*n* (%)
Gender	
Male	2 (18)
Female	9 (82)
Age range	
21–24	7 (64)
25–27	4 (36)
Level of education	
Bachelor’s degree	10 (91)
Master’s degree	1 (9)
Religion	
Islam	8 (72)
Christian	3 (28)
Ethnicity	
Ambon	1 (9)
Batak	1 (9)
Jawa	3 (28)
Minang	3 (28)
Nusa Tenggara	1 (9)
Sunda	2 (17)

### Problems and solutions

Thematic data analysis provided two broad areas/themes: (1) Interviewing problems and solutions and (2) technical problems and solutions. A distinction can also be made between (1) problems in interviewing encountered by interviewers and (2) problems in interviewing encountered by respondents (as perceived by interviewers).

### Problems in the interviewing process encountered by interviewers

#### Recruitment of respondents

This theme concerned any problem related to finding a respondent and receiving his/her consent to participate in the study. Table 2 shows that the most frequently mentioned problem was to find suitable respondents. Obstacles identified were time and activity of the respondents, and local government permission to collect data.

**Table 2:** Problems of interviewers

No	Problems	Frequency^a^
1	Recruiting respondents	28
a	Finding respondents	24
b	Acquiring participation consent	4
2	Interview process	24
a	Conducting protocol steps (TTO and DCE)	15
b	Dealing with respondents	4
c	Interviewer personal issues	5

^a^Number of times the related problem was mentioned by interviewers

An interviewer highlighted time and activity of respondents as her most frequent obstacles: “Sometimes it was difficult to make a schedule and arrange an appointment that matched the respondent’s free time, considering that the interview usually takes more than 1 h.” Another interviewer wrote: “When an appointment is already agreed but a respondent asks to change the day or time of interview.”

In Indonesia, it is common to ask the local official and unofficial authorities for permission to undertake any kind of research. The time and effort required to obtain such permission to collect data was also a problem for interviewers. “Some rural areas that we contacted earlier asked for a formal permission letter from the kecamatan (district) office, then permission from the kelurahan (sub-district), and from the head of the desa (village). This is the formal procedure.”

Some respondents with specific characteristics were also difficult to find, especially ethnicities other than Jawa and Sunda in the rural areas and respondents aged over 50. “To find respondents with difficult-to-find characteristics, such as ethnicities non-Jawa and Sunda or respondents aged more than fifty.”

#### Interview process

This theme was defined as any problem related to conducting the data collection process, including following the protocol and dealing with the respondent’s and interviewer’s personal issues. Most interviewers mentioned at least one problem within this theme. To explain the procedure and practice section of the EQ-VT protocol was the most frequently mentioned problem. This was not only the case for lower-level education respondents: as one interviewer wrote “It took a long time to explain TTO to lower-level education respondents,” but also for some of the higher-level education respondents. “Because my respondents are usually people with a higher level of education, they are more critical with respect to some health states that are illogical in their opinion, such as ‘have no difficulty to walk’ but ‘can’t take a bath.’”

Another problem was to deal with physical and psychological issues that resulted from the long duration of the interview. Respondents became tired and bored during the TTO and DCE exercises. “Respondents usually became tired after the Feedback Module.”

Four interviewers admitted personal issues in conducting the interviews, including becoming bored during the interview session themselves, carelessness, not having the confidence to build good rapport and confusion about how to explain the instructions and questions in a well-understood way. “At the beginning it was difficult for me to explain to the respondents about this research because I didn’t know the best way and tricks to explain it better.”

### Problems in interviewing encountered by respondents (as perceived by interviewers)

#### Participation

This theme comprised negative thoughts and feelings which were expressed regarding participation in the study during the explanation of informed consent. Five interviewers reported evidence of this, including respondents: (1) suspecting the interviewer of deception, or that this was not real research, (2) being afraid of the possibility his/her answers would be recorded on a recorder tape and (3) hesitating to write their real name and address. “Some respondents were hesitant because they were afraid that this research was a fraud or had a hidden agenda.”

#### Interview process

This theme was defined as problems faced by respondents during the interview session, as perceived by the interviewers. As shown in Table 3, cognitive difficulty was the most frequently mentioned issue, with 8 interviewers mentioning respondent cognitive difficulties during interviews, especially the difficulties in differentiating between the different dimensions and levels of EQ-5D-5L and the different questions in TTO and DCE. One interviewer wrote “Some respondents had difficulty to differentiate between the levels of health states (no problem until severe).”

**Table 3:** Problems of respondents (as perceived by interviewers)

No	Problems	Frequency^a^
1	Participation	8
2	Interview process	68
a	Cognitive difficulties	30
b	Emotional difficulties	7
c	Physical difficulties	4
d	Religious beliefs	17
e	Presence of others	10

^a^Number of times the related problem was mentioned by interviewers

The second problem most frequently mentioned by the interviewers was strong religious beliefs and respect for life that interfered with how the respondent should follow the data collection process. The majority of interviewers encountered this problem. Some respondents believed that every word they said was a prayer, so it was difficult for them to choose instant death in a TTO question. One interviewer wrote “For respondents who have strong religious beliefs, they believed that every word they said was like a prayer. Some refused to continue their participation because of the option of Instant Death in TTO. Others preferred not to choose Instant Death even though the health state in the question was really bad. They believed that there would be someone else who would help them and because they believed that life and death were in God’s hands.” Other respondents had a strong preference for life and did not want to sacrifice a year or only sacrifice 6 months to a maximum of one year for any TTO question. “Some respondents had a strong belief that no matter how bad the health status was, they would not sacrifice any year. They believed that in that bad situation they could still do something useful.”

For some respondents, their physical condition interfered with their efforts at completing the interview. “For some respondents aged more than fifty years old, I had to read the feedback module section for them because their eye function was already reduced and it was difficult for them to read a screen full of small letters.” Boredom and fatigue were also experienced by some respondents when completing the TTO and DCE tasks. The problems came not only from the respondents themselves but also from the presence of others, whether or not they knew these people. Their presence was distracting the respondents from the task or interfered with how they answered or wished to answer the questions. “One of my respondents was interviewed in her house in the presence of her little daughter in the room. When she selected the instant death choice in one of the TTO questions, her daughter displayed a shocked reaction that resulted in the respondent changing her answer.”

### Solutions applied by interviewers

#### Recruitment of respondents

This theme concerns the efforts made to solve the problems of finding respondents to participate in the study. There were 3 main solutions that the interviewers applied: (1) take into account a variety of factors which would enable suitable respondents to be found, (2) expand networks and (3) explain the study thoroughly (see Table 4).

1. Interviewers considered specific characteristics with respect to respondents in finding them for the quota sample. Two main factors to be considered are time of availability and areas where many potential respondents lived. Since a respondent’s time was the obstacle most frequently mentioned by interviewers, arranging appointments at times when respondents had the most free time was the most frequently employed strategy. People with fixed daily schedules, housewives and people who worked as merchants in tourist spots were usually chosen to be interviewed. Weekends, evening and lunch time were preferable times to conduct interviews for interviewers. “Conduct an interview at lunch hour or after office hours when the respondent is free.”
2. Interviewers used their networks to find suitable respondents, such as their relatives, friends and even the respondents themselves. “Contact families and friends who might have access to people with specific characteristics, such as people with lower-level education or females aged above 50 years old.”
3. Interviewers persuaded respondents to participate in the study by utilizing the local government permission procedure and explaining thoroughly the goals and benefits of the study. For some respondents, a letter from the local authority was enough for them to participate. For other respondents, explaining that the results of the study would be used by the Indonesian government and other stakeholders for the benefit of the Indonesian people in the future encouraged them to participate. “Explain slowly about the goals of this research in more concrete words, such as: this survey is about health and will measure the perceptions of Indonesian people about health and health problems. The results will be used by the Indonesian government to create useful health policies. So your participation is really valuable for the improvement of the healthcare system in Indonesia.”

**Table 4:** Solutions applied by interviewers

No	Solution	Frequency^a^
1	Recruiting respondents	49
a	Selective search	22
b	Using networks	6
c	Explaining in detail	21
2	Process of interview	72
a	Developing self	14
b	Stimulating respondent	58

^a^Number of times the related solution was mentioned by interviewers

#### Interview process

This theme comprised any effort to solve respondents’ difficulties during data collection by stimulating them using various means, and by developing interviewer’s personal skills. The majority of interviewers helped their respondents to complete interviews by putting extra effort into the process of interviewing. This could involve: (1) giving additional explanation or rephrasing the explanation in words that were easier to understand, (2) asking the respondent to imagine concrete examples of the question, (3) guiding the respondent to look in detail at each health state, (4) reassuring the respondent about the implications of his/her answer. For example, to help respondents who had difficulty in comparing life A and life B in the DCE exercise, an interviewer did the following: “I helped my respondents to choose by asking them to compare each dimension in life A and life B, not just to read it quickly and give an answer.” With respect to the problem of religious beliefs, further explanation concerning the nature of the TTO exercise, i.e., that this should be considered as a cognitive task, was quite effective in reassuring some respondents. “I explained that this was research concerning his opinions on a number of health states, not his prayer to God. My respondent then understood and provided appropriate answers based on his opinions.”

Some interviewers chose to focus on their relationship with the respondent by talking about other things (for instance family or work), encouraging him/her to continue and praising him/her after finishing each part of the interview. “Talk about other things first before going into an interview, usually about the respondent’s daily life.” In addition to working/training with their respondents during interviews, some interviewers also developed their own skills in order to improve the quality of their interviews, by additional reading and practice in order to get used to the protocol and the software as quickly as possible. “After I get used to this EQ-VT protocol and guideline, I can explain it better to the respondents.”

### Technical problems and solutions

Technical problems were defined by any problems faced by interviewers and respondents that were related to technical tools used in the study, including hardware (laptop), software (EQ-VT software, www.qol-id.org, Mozilla Firefox, Teamviewer) and internet connections. As shown in Tables 5 and 6, three themes emerged with respect to technical problems.

**Table 5:** Technical problems

No	Problems	Frequency^a^
1	Hardware	9
2	Software	13
3	Internet connection	5

^a^Number of times the related problem was mentioned by interviewers

**Table 6:** Solutions of technical problems

No	Solutions	Frequency^a^
1	Independent problem solving	16
a	Laptop	4
b	Software	8
c	Internet connection	4
2	Dependent problem solving	9
a	Laptop	1
b	Software	7
c	Internet connection	1

^a^Number of times the related solution was mentioned by interviewers

#### Hardware problems

Four interviewers reported having problems with their laptops during data collection. For 3 interviewers, these related to short battery life, limiting the number of respondents seen in one day to no more than 2, or they had to find an interview location that provided an electric socket. Another interviewer had limited random access memory (RAM) in her laptop that made it work more slowly than usual.

#### Software problems

Five interviewers had problems with software. They had to register their respondents on a Web site created specifically for the Indonesian valuation study, and received a respondent code, which they used as external ID in EQ-VT software. The problems they encountered varied, such as difficulties in registering a respondent, accessing the offline ULP and uploading the interview data. “I can’t access the EQ-VT offline ULP in my laptop so I couldn’t conduct the interview.”

#### Internet connection problems

This problem is related to the availability and functionality of connection to the internet during the Indonesian valuation study. One interviewer wrote, “Before starting an interview, I have to register my respondent online in order to get a respondent code. If I have to register a respondent that I find without any previous appointment, this online registration becomes a problem when my phone signal is weak or there is even no connection at all.”

#### Technical solutions

This theme comprises the efforts of interviewers to solve any problems related to tools used in the study (laptop, software, network) with and without help from others. Tables 5 and 6 show the two sub-themes that emerged from the analysis.

#### Independent problem solving

This sub-theme was defined by the efforts of the interviewer to solve problems related to tools used in the study independently without help from others. “Find a place to conduct the interview where electrical socket available” was one interviewer’s effort to overcome a laptop battery problem. Another interviewer wrote, “I make sure my laptop is fully charged before I conduct an interview, especially the offline EQ-VT software. I also regularly upload my interviews so that my Firefox will download new questionnaires every day.” To cope with network problems, another interviewer always took along a mobile internet modem.

#### Dependent problem solving

This sub-theme involved help from others. Interviewers asked for help from their fellow interviewers when this related to laptop and network problems, and from the EuroQol Office when it concerned EQ-VT software problems.

### Improvement of interviewers’ performance

From the 286 potential respondents asked to participate, 266 respondents were interviewed (93% response rate). Table 7 shows that all seven indicators of interviewers’ performance—monitored in the Quality Control process—in conducting EQ-VT interviews were found to be significantly improved (post-retraining scores, *p* < 0.05). For example, the percentages of flagged interviews, which were the main indicator of quality, showed a large improvement from 59.2 to 3.6% between pre-retraining and post-retraining. Moreover, the wheelchair explanation moves increased from 14.66 to 58.98 and flagged TTO interview time decreased from 15.3 to 1.2% which is indicators of more engagement and less hurry on the part of the interviewers while preparing the participants through wheelchair example and conducting the 10 TTO tasks.

**Table 7:** Results of Wilcoxon signed-rank test of indicators of performance

Indicator	Pre-retraining	Post-retraining	*p* value
Number of interviews	98	168	
Number of health states given a zero TTO value	275 (28.1%)	65 (3.9%)	0.003*
Mean wheelchair explanation moves	14.66	58.98	0.003*
Inconsistencies flagged	6 (6.1%)	2 (1.2%)	0.046*
Wheelchair explanation time flagged	39 (39.8%)	1 (0.6%)	0.003*
TTO interview time flagged	15 (15.3%)	2 (1.2%)	0.026*
Wheelchair lead time flagged	20 (20.4%)	1 (0.6%)	0.027*
Flagged interviews^a^	58 (59.2%)	6 (3.6%)	0.005*

The amount of “flags” based on corresponding indicator of quality

* *p* < 0.05

^a^An interview can have more than one flag; therefore, the column cannot be summed to a total

## Discussion

This study enlisted sampling and technical problems encountered by the Indonesian EQ-5D-5L valuation study interviewers. Moreover, the substantial quality issue with the first 98 interviews has been described. A comprehensive strategy to acquire suitable respondents, including involving personal and formal networks and optimizing interview times according to the availability of respondents, was implemented by the interviewers to overcome sampling problems. Technical problems were dealt with using the capabilities of the interviewers to improvise on a local level and technical support from the EuroQol Group office at a central level. To improve the quality of interviews, a retraining program and subsequent feedbacks based on the quality control (QC) report were implemented which lead to good quality data.

The first problem encountered by interviewers was to find respondents who were willing to participate in the study. Some participation problems have been mentioned in the literature [21, 22], although not with particular reference to TTO and DCE exercises. For example, some respondents were anxious about their participation in our research. This might happen because individuals realized that they would be asked to answer personal questions [23] or had minimal knowledge of what would happen [24]. Being well-prepared and having a good ability to establish rapport as an interviewer are known to be essential to reduce respondents’ levels of anxiety [24]. Respondents also prefer interviewers that they know [25], have similar characteristics to them [26], and use their preferred language [27]. Solutions applied by the interviewers in this study, such as involving personal networks and explaining informed consent in simple, easy-to-understand words, were effective in coping with the problem of finding respondents. Another problem was to match a respondent’s availability with the interviewer’s schedule in terms of time and place. Choosing a time most suitable for the respondent to be focused on the interview and a comfortable location that is convenient are vital in ensuring an optimal interview process [24]. It turned out that some interviewers specialized in groups of subjects, such as the young, the elderly, or the working population. When quota sampling is stratified per interviewer, it is not possible to explore this specialization. Hence, we allowed interviewers to specialize in categories of respondents, until the category was full at the aggregate sample level. Evidently, given possible interviewer effects, the situation should be avoided that interviewers be solely responsible for filling one category of subjects. In our study, this exclusive interviewing was not the case.

The second problem was to conduct an interview that followed the essential parts of the protocol, but was adaptive enough to help respondents complete the interview. Respondents experienced various difficulties, from cognitive and emotional to physical. To solve this problem, interviewers’ interviewing and communication skills play important roles [24]. This study’s interviewers had some training in interviewing skills during their Bachelor’s degrees and/or followed a 1-day interviewing skills workshop held by the first author (FDP) before the start of the valuation study. Attentive listening and ability to direct interviews using various means are essential to keep respondents focused on their tasks [28]. Asking questions to stimulate the thought process, especially in the TTO section of the interview, and giving examples that closely relate to a respondent’s experiences are effective interview tools [26]. All of this was confirmed in our study.

This study found that the interviewers struggled to implement the standard valuation protocol for an EQ-5D-5L valuation study, based on the first QC report. A similar problem was also reported by Papadimitropoulos et al. [10] in the United Arab Emirates, in which their interviewers were from a market research agency. Their recommendation was to train academic researchers in health state valuation and state preference methods in order to increase the availability of skillful interviewers. Tasks such as TTO and DCE have high cognitive burdens. The presence of experienced interviewers is essential in ensuring the validity of such tasks. This means that the training of interviewers plays an important role in assuring data quality [19]. To meet these criteria, we hired interviewers with academic backgrounds related to the topic of quality of life such as Psychology, Management, Development Communication and Economics. To equip interviewers with the relevant knowledge of health state valuation and stated preference methods, we conducted 1-day training sessions before commencing the study where interviewers learned about the basic concept of quality of life and its measurement. They also learned about how to value quality of life, in this case by using the TTO and DCE approaches. Time and tools for interviewers were provided to practice using EQ-5D-5L valuation software and protocols. Nevertheless, we still encountered the same problem as in UAE regarding protocol compliance. When the initial training proved insufficient to guarantee the expected quality of the data, a retraining program was conducted. This program and a series of QC reports and feedback led to higher compliance by the interviewers to the protocol. This was demonstrated in the QC report by significantly less flags (indicating quality problems), less zero values, less flagged inconsistencies, less flagged TTO time, less flagged wheelchair explanation time and more wheelchair lead time and wheelchair moves. We can expect that this protocol compliance problem will emerge in any valuation study regardless of the interviewers’ background characteristics; hence, the similar solution should be implemented: utilization of quality control (QC) report through training and consistent feedback.

Indonesia is a country where religious belief plays a big role in its residents’ lives [29]. Religious belief and respect for life also appeared to influence respondents’ perceptions of the TTO questions, especially with respect to “instant death” and “worse-than-dead.” [10, 30]. It was believed that “words are prayer” which resulted in hesitation or even rejection in choosing the instant death and worse-than-dead answers. Some respondents even withdrew from the interview during the worse-than-dead explanation in the wheelchair example due to this belief. Similar problem was reported by researchers from United Arab Emirates (UAE), Malaysia and Singapore [10, 30, 31]. This issue is less problematic during valuation studies in more secular countries in the western hemisphere such as UK and The Netherlands [2, 5]. Interviewers have to ensure the cultural safety of research participants, i.e., by taking their religious beliefs into account [32]. Therefore, we expect similar issue will happen during valuation study in the countries where Islam is the majority religion or in the Islamic subset of a population. A solid rapport and various strategies, such as further explanation, rephrasing the explanation in words that were easier to understand, and stressing the goals and benefits of the study to encourage respondents to give their cognitive opinions instead of emotional responses, proved effective enough to handle this situation.

## Strengths and limitations

This is the first study using Quality Control report to optimizing performance of interviewers and the quality of the data collected in a valuation of EQ-5D-5L. Furthermore, this study comprehensively describes the problems and solutions of interviewers and respondents in performing TTO and DCE tasks, as well as technical and methodological issues. Finally, several possible solutions and their impact on the quality of the interviews are also provided. The lessons learned from this study could serve as examples discussed in the initial training of EQ-5D-5L valuation study.

Several limitations of this study should be considered. First, the study shows that a QC report was an important factor in optimizing performance at interviews and the quality of the data collected. However, this was a formal process and focused on several objective indicators, such as consistency, duration, which did not take into account what was actually said, let alone the nonverbal interaction between interviewer and respondent. Nevertheless, it was the first step in getting a grip on and improving the interview process.

Second, respondent recruitment might raise questions about the objectivity/representativeness of the study sample since one of the solutions employed was to use personal networks related to the respondents. This might have entailed some bias in terms of interdependent data collection. However, since this was done in order to find respondents to fit into the missing categories in the quota sample (for example those with low education and the relatively old), it was judged that this was a lesser problem than insufficiently filled categories in the quota sampling. This was because the quotas were determined on those variables that were seen beforehand to be important as defining representativeness. In that respect, we have constructed a representative sample. Nevertheless, a limitation could be that the sample might be in part the networks of the interviewers. It remains to be seen whether this is a problem.

Third, it is not known what kind of problems were associated with those who did not want to participate, i.e., 20 people out of 286 asked to participate.

Fourth, the classification of urban and rural in this study was based on the governmental administrative definition. During the process, it was found that some areas classified by municipal administrations (kabupaten) as rural in no way represented the characteristics of a rural area. They were Jatinangor where Universitas Padjadjaran is located, and Depok Sleman where Universitas Gadjah Mada is located. Respondents from these two areas were, therefore, categorized as urban respondents instead of rural.

Fifth, the interviewers’ improvement analysis was based upon data from 266 respondents, 25% of the targeted number of respondents. It may thus be wondered how representative the problems discovered were for the complete sample, as this 25% were in particular young, relatively well-educated and urban respondents. Of the 266 respondents, only 39 elderly respondents (14.6%), 5 low-educated respondents (1.8%) and 53 rural respondents (19.9%) were interviewed. Hence, it could be stated that the interviewers started with an easy, smart and “well behaved” sample. One can question whether this a problem, or an advantage. It can be seen as a problem, as interviews with “difficult” subjects were less frequently undertaken. On the other hand, it makes sense to learn the interview skills first in a relatively “easy” sample, and then to undertake the more difficult interviews later, when the interviewer would be well trained. Indeed, we would recommend commencing with the easy interviews and moving on to the more difficult.

Sixth, all interviews were conducted in 3 cities on Java island, even though some ethnicities interviewed were not originally from Java (e.g., Sumatera, Bali, Madura and Sulawesi). We do not know whether different problems, such as language barrier, would emerge if the interviews were to be conducted in the home towns of these ethnicities.

Seventh, the interviewers were asked about their problems and solutions during the interview by the researchers, who were also the person who hired them and evaluate their quality of work. This would potentially influence the interviewers to provide more positive answers compared to the actual situation in the field. However, since we perceived the discussion during feedback sessions as positive, open and equal, we think that we have been as careful as possible in that respect. The interviewers’ feedback to the researchers in the end of valuation study data collection showed the same conclusion.

Lastly, the retrospective character of the study, in which the interviewers received the questionnaire at a later date, might have been liable to recall bias and led to the omission of some information.

## Recommendations

Given the limitations of this study, there are some suggestions for future research. Regarding the method of controlling the quality of interviewers’ performances, it would be better to put the interviews from a small representative sample of interviewers before and after the retraining program on video to establish which elements of the retraining yielded improvement. Interviewers should be asked to note the problems occurring while interviewing immediately instead of at a later date. Recruiting more elderly, more lower-educated and more rural respondents at the outset could give more information about the specific problems of these categories of respondents. In order to avoid disappointment and frustration in the research team, an interviewer’s first 10 interviews could be used as a pilot to measure quality, to provide feedback and to ensure good quality subsequently. Evidently such a pilot phase for each interviewer would increase costs, but it would reduce the costs associated with modeling low-quality data. This leads to a recommendation to limit the number of interviewers, in order to optimize the quality per interview. It is further recommended that information regarding the problems and solutions encountered during valuation studies should also incorporated in future interviewer training manuals.

## Conclusion

This study has identified several sampling issues and technical problems in conducting the standard EQ-5D-5L valuation protocol. Moreover, substantial quality issues in the interviews process have been described. Sampling problems could be overcome by a comprehensive search strategy involving broader networks and optimization of interview times for respondents. Quality issues in the interview could be dealt with using feedback from the QC report, a comprehensive training program, and increased supervision at the start of and during the study. If the interviewers were to become more engaged in the research, the quality of the interviews should improve. We recommend limiting the number of interviewers and relying on academically skilled interviewers who could be expected to fully understand the research aims. Using a quality control feedback module, organizing continuous feedback sessions, and accepting a pilot phase for each interviewer, should help to optimize the quality of data collection.
